# Worn-Out Hospital Officials

**Published:** 1913-12-06

**Authors:** 


### THE EDITOR'S LETTER-BOX.

### Worn-Out Hospital Officials: The Pension Question.

*To the Editor of* The Hospital.

Sir,—A few months back there appeared in your columns something more than a hint that those who had grown grey in hospital service should be replaced by younger and more efficient persons. I know I felt the suggestion at the time I read it, for it was a case of the cap fitting.

I quite admit that there are times when the interests of an institution may suffer through members of its staff hanging on to their positions too long, and that in many cases the substitution of younger men or women, as the case may be, would be advantageous, for it must be acknowledged that length of service is apt to incline one to become groovy, and resent innovations in the shape of new ideas and up-to-date methods.

Having now served as a hospital secretary and superintendent for over thirty years, I recognise this fact, and though experience counts for something in hospital administration, still I feel that a more capable man might be found to take my place, and, at all events, one possessing more enthusiasm and energy for work than I can now lay claim to. But in one respect I'm like the steward in the parable, for I cannot dig and I won't beg, while I've never received a large enough salary to enable me to provide for old age, so that at this late hour of my life (the mid sixties) I find myself (largely owing to unfortunate investments and family troubles) with a mortgaged whole-life insurance policy as my sole monetary asset. Hence, with three lives dependent on me, there is nothing for it but to stick to my present position as long as I can, trusting that when my faculties become impaired a merciful Providence may grant me a quick and happy despatch, for I am not among those who pray to be delivered "from sudden death."

In this part of the world the Government will not permit any gratuity or retiring allowance to be paid to the superannuated employees of charitable institutions, such luxuries being reserved for "swagger" officials who have big screws and soft billets. Thus, for those circumstanced like myself, the financial outlook is decidedly gloomy.—Yours, &c., Hospital Secretary.

Australia, October 30, 1913.

